# Complete genome sequence of *Diaporthe vaccinii* Shear, a fungal isolated from blueberry

**DOI:** 10.1128/mra.00001-25

**Published:** 2025-08-27

**Authors:** Xiufen Wang, Rong Lei, Xin Li, Limei Li, Xinyi Wang

**Affiliations:** 1 Technology Center of Dalian Customs District, Dalian, China; 2 Chinese Academy of Inspection and Quarantine, Beijing, China; 3 Jilin Provincial Academy of Forestry Science 468880, Changchun, China; 4 School of Life and Health, Dalian University 74547, Dalian, China; University of California Riverside, Riverside, California, USA

**Keywords:** *Diaporthe vaccinii*, complete-genome sequence

## Abstract

The complete genome sequence of *Diaporthe vaccinii* Shear was reported using combined Oxford Nanopore and Illumina sequencing. The hybrid genome comprises 64 contigs with a total length of 60.2 Mb and a GC content of 48.3%.

## ANNOUNCEMENT

*Diaporthe vaccinii* Shear (strain CBS 118571), deposited in the Westerdijk Fungal Biodiversity Institute (CBS-KNAW, Utrecht, Netherlands) following its original isolation from blueberry (*Vaccinium corymbosum*) in Michigan, USA by G.C. Adams represents a destructive plant pathogen causing substantial economic losses in commercial *Vaccinium* crops (1) through its necrotrophic pathogenesis manifesting as sequential twig blight, stem canker, dieback, leaf chlorosis, and postharvest fruit rot (2). To elucidate its molecular virulence determinants, we implemented a hybrid sequencing strategy combining Oxford Nanopore Technologies (ONT) with Illumina short-read sequencing to obtain a high-contiguity *de novo* genome assembly.

A piece of *D. vaccinii* mycelium colonies was put on a solid PDA plate and grown at 20°C with a 12 h-dark/12 h-light cycle for 7–10 days. Freshly propagated mycelia and spores were aseptically harvested with an inoculation needle for genomic DNA isolation using the DNeasy Plant Pro Kit (Qiagen, Germany), whereas total RNA integrity extracted with an RNAprep Pure Plant Plus Kit (Tiangen, China) was verified through an Agilent 2100 bioanalyzer (Agilent, USA) employing RNA 6000 Nano reagents. ONT long-read sequencing libraries were prepared through mechanical DNA fragmentation (Covaris g-Tube, Cat# 520079) coupled with nucleic acid purification via Beckman AMPure XP Reagent (Cat# A63881), followed by enzymatic end-pair and dA-tailing using NEBNext FFPE Repair Mix (Cat# M6630) and Ultra II End Repair/dA-tailing Kit (Cat# E7546), respectively. Ligation of ONT-specific adapters (SQK-LSK109) employed NEBNext Quick Ligation Reaction Buffer (Cat# B6058) and NEB T4 DNA Ligase 2 M U/mL (Cat# M0202), and size selection for filtering out fragments shorter than 3 k bp was conducted, prior to loading 600 ng library onto a PromethION P48 platform equipped with R9.4.1 Flow Cell. Parallel Illumina DNA-Seq libraries were constructed through NEBNext dsDNA Fragmentase (Cat# M0348)-mediated digestion of 1 µg genomic DNA using the ExCell Bio Library Construction Kit (NuGEN, San Carlos, CA, USA), followed by Illumina NovaSeq 6000 sequencing with PE150 and PE250 format. The RNA-seq library was prepared using the TransNGS Stranded RNA-Seq Library Prep Kit (TransGen, China), followed by quality control on an Agilent 2100 Bioanalyzer and Illumina NovaSeq 6000 sequencing with PE150 format. Base calling of ONT raw signals utilized Guppy v6.5.7 (dna_r9.4.1_450bps_hac model) (3) with PoreChop v0.2.4 adapter trimming (4). Raw Illumina DNA-Seq reads (PE150: 13,896,344,100 bp, PE250: 896,775,500 bp) were processed through Skewer v0.2.2 (5) (minimum mean-quality threshold 20) to yield clean data (PE150: 11.75 Gb, and PE250: 0.64 Gb). A total of 8.25 Gb Illumina RNA-Seq clean reads were also produced to facilitate downstream genome annotation.

Hybrid assembly of *D. vaccinii* CBS 118571 via MaSuRCA v3.3.7 (6) produced a 60.193 Mb draft genome (64 contigs, N50 = 1.70 Mb). Burrows-Wheeler Alignment tool v0.7.13-r1126 (7) demonstrated 97.99% of Illumina reads successfully mapping to the assembly. Long-read alignment using minimap2 2.24-r1155-dirty (8) revealed 89.92% of ONT reads mapping. Genome completeness assessment through BUSCO v4.1.4 (9, 10) utilizing the fungi_odb10 data set identified 99.3% of conserved fungal orthologs as complete, confirming exceptional assembly continuity (Table 1).

**TABLE 1:** Genome features of *D. vaccinii* CBS 118571

Features	Strain CBS 118571
ONT long reads (sequencing depth)	7.864 Gb (~ 130.7 X)
Average read length	15,336.35
Reads N_50_	24,998
Maximum read length	133,213
Illumina short reads (sequencing depth)	14.79 Gb (~ 245.8 X)
Estimated genome size	58.82 Mb
Estimated genome repeats	14.15%
Estimated genome heterozygosity	0.08%
RNA-seq	8.25 Gb (~ 137.1 X)
Assembly size	60.19 Mb
Contig number	64
Contig N_50_	1,704,828
Contig L_50_	11
Average contig length	940,511.64
Maximum contig length	4,440,232
GC content	48.3%
Repeat sequence	10.14%
BUSCO fungal integrity	99.3% [S:98.9%, D:0.4%] F:0.3%, M:0.4%
Illumina reads mapping rate (by BWA) ONT long reads mapping rate (by minimap2)	97.99% 89.92%

## Data Availability

The whole-genome data of *D. vaccinii* CBS 118571 presented here have been deposited at GenBank under the accession number JBCEDN000000000. The associated BioProject, and BioSample accession numbers are PRJNA1091205, SAMN40589633, respectively. The SRA accession number of ONT reads, Illumina PE150 reads, Illumina PE250 reads, and RNA-Seq PE150 reads are SRR29094102, SRR29094105, SRR29094104, and SRR29094103, respectively.

## References

[1] Farr DF, Castlebury LA, Rossman AY. 2002. Morphological and molecular characterization of Phomopsis vaccinii and additional isolates of Phomopsis from blueberry and cranberry in the eastern United States. Mycologia 94:494–504. doi:10.2307/3761783. 21156520

[2] Jeger M, Bragard C, Caffier D, Candresse T, Chatzivassiliou E, Dehnen-Schmutz K, Gilioli G, Grégoire J-C, Jaques Miret JA, MacLeod A, Navarro MN, Niere B, Parnell S, Potting R, Rafoss T, Rossi V, Urek G, Van Der Werf W, West J, Winter S, Gardi C, Mosbach-Schulz O, Koufakis I, Van Bruggen A, EFSA Panel on Plant Health (PLH). 2017. Pest risk assessment of Diaporthe vaccinii for the EU territory. EFSA J 15:e04924. doi:10.2903/j.efsa.2017.4924 32625637 PMC7010004

[3] Wick RR, Judd LM, Holt KE. 2019. Performance of neural network basecalling tools for Oxford Nanopore sequencing. Genome Biol 20:129. doi:10.1186/s13059-019-1727-y 31234903 PMC6591954

[4] PoreChop (v0.2.4). 2018 https://github.com/rrwick/Porechop.

[5] Jiang H, Lei R, Ding SW, Zhu S. 2014. Skewer: a fast and accurate adapter trimmer for next-generation sequencing paired-end reads. BMC Bioinformatics 15:182. doi:10.1186/1471-2105-15-182 24925680 PMC4074385

[6] Zimin AV, Marçais G, Puiu D, Roberts M, Salzberg SL, Yorke JA. 2013. The MaSuRCA genome assembler. Bioinformatics 29:2669–2677. doi:10.1093/bioinformatics/btt476 23990416 PMC3799473

[7] Li H, Durbin R. 2009. Fast and accurate short read alignment with Burrows-Wheeler transform. Bioinformatics 25:1754–1760. doi:10.1093/bioinformatics/btp324 19451168 PMC2705234

[8] Li H. 2018. Minimap2: pairwise alignment for nucleotide sequences. Bioinformatics 34:3094–3100. doi:10.1093/bioinformatics/bty191 29750242 PMC6137996

[9] Simão FA, Waterhouse RM, Ioannidis P, Kriventseva EV, Zdobnov EM. 2015. BUSCO: assessing genome assembly and annotation completeness with single-copy orthologs. Bioinformatics 31:3210–3212. doi:10.1093/bioinformatics/btv351 26059717

[10] Seppey M, Manni M, Zdobnov EM. 2019. BUSCO: assessing genome assembly and annotation completeness. Methods Mol Biol 1962:227–245. doi:10.1007/978-1-4939-9173-0_14 31020564

